# Robot-assisted transthoracic hybrid esophagectomy versus open and laparoscopic hybrid esophagectomy: propensity score matched analysis of short-term outcome

**DOI:** 10.1007/s00423-022-02667-6

**Published:** 2022-09-06

**Authors:** Therese Reinstaller, Daniela Adolf, Eric Lorenz, Roland S. Croner, Frank Benedix

**Affiliations:** 1 Department of Surgery, University Hospital Magdeburg, Leipziger Strasse 44, 39120 Magdeburg, Germany; 2 StatConsult GmbH, Halberstädter Strasse 40a, 39112 Magdeburg, Germany

**Keywords:** Robot-assisted hybrid esophagectomy, Minimally invasive hybrid esophagectomy, Esophageal cancer, Postoperative outcome

## Abstract

**Purpose:**

Minimally invasive en-bloc esophagectomy is associated with a reduction of postoperative morbidity. This was demonstrated for both total minimally invasive and hybrid esophagectomy. However, little is known about any benefits of robotic assistance compared to the conventional minimally invasive technique, especially in hybrid procedures.

**Methods:**

For this retrospective study, all consecutive patients who had undergone elective esophagectomy with circular stapled intrathoracic anastomosis using the open and the minimally invasive hybrid technique at the University Hospital Magdeburg, from January 2010 to March 2021 were considered for analysis.

**Results:**

In total, 137 patients (60.4%) had undergone open esophagectomy. In 45 patients (19.8%), the laparoscopic hybrid technique and in 45 patients (19.8%), the robot-assisted hybrid technique were applied. In propensity score matching analysis comparing the open with the robotic hybrid technique, significant differences were found in favor of the robotic technique (postoperative morbidity, *p* < 0.01; hospital length of stay, *p* < 0.01; number of lymph nodes retrieved, *p* = 0.048). In propensity score matching analysis comparing the laparoscopic with the robotic hybrid technique, a significant reduction of the rate of postoperative delayed gastric emptying (*p* = 0.02) was found for patients who had undergone robotic esophagectomy. However, the operation time was significantly longer (*p* < 0.01).

**Conclusions:**

En-bloc esophagectomy using the robotic hybrid technique is associated with a significant reduction of postoperative morbidity and of the hospital length of stay when compared to the open approach. However, when compared to the laparoscopic hybrid technique, only few advantages could be demonstrated.

## Introduction

The global incidence of esophageal carcinoma, especially of adenocarcinoma of the esophagogastric junction (AEG tumors), is steadily rising [1, 2]. This can mainly be attributed to the worldwide increase of obesity and growing numbers of patients with gastroesophageal reflux disease [3, 4]. The current standard of care in Germany for patients with locally advanced, resectable carcinoma of the middle and lower third of the esophagus without distant metastasis comprises a neoadjuvant therapy followed by surgical resection [5]. Oncologic en-bloc esophagectomy can be regarded as one of the most complex and challenging surgical procedures. Despite significant improvements in perioperative management of patients, these procedures remain associated with a relevant morbidity and mortality even today [6]. The most frequent and most feared procedure-related complications are anastomotic leaks and pulmonary complications during the postoperative course. Morbidity and mortality rates following several surgical and oncological procedures have significantly improved during the last decades due to the advancement of surgical techniques. Especially the introduction of minimally invasive techniques resulted in many advantages for the patients, i.e., a lesser operative trauma, faster recovery, less pain, and a reduction of the ICU and hospital length of stay [7]. A randomized controlled trial (TIME trial) also confirmed the superiority of the minimally invasive approach in complex esophageal resections. Patients who had undergone minimally invasive en-bloc esophagectomy experienced significantly less pulmonary complications during the postoperative course compared to those who had received an open resection [8]. Interestingly, in another randomized controlled trial, this was also confirmed for hybrid esophagectomy (abdominal part: laparoscopically; thoracic part: open). The hybrid approach was associated with a significant reduction of postoperative morbidity, especially a reduction of pulmonary complications when compared with open resection (MIRO trial) [9]. The results of this latter study might suggest that patients mainly benefit from the reduction of abdominal trauma during the procedure. It is currently unclear whether complete minimally invasive esophagectomy (laparoscopic/thoracoscopic approach) is superior when compared with the hybrid procedure. During the last decade, a growing interest in robotic techniques for oncologic gastrointestinal procedures can be observed. Today, many centers worldwide are using robotic assistance for several surgical procedures. This trend also holds true for esophageal resections. Several surgeons have established robot-assisted esophagectomy during the last years, both as full robotic and hybrid procedures. In a randomized controlled trial, it was demonstrated that the use of robotic assistance also provides significant benefits for patients undergoing en-bloc esophagectomy when compared to the open approach (ROBOT trial) [10, 11]. Today, little is known about whether full robot-assisted esophagectomy is superior to conventional total minimally invasive esophagectomy. There are two studies that show potential benefits when using the robotic technique [12, 13]. However, there are nearly no studies that compare conventional and robotic hybrid esophagectomy. Thus, it is not clear whether patients who undergo hybrid esophagectomy will benefit from the use of robotic aid, and if the higher procedural costs are thus counterbalanced. The main focus of the present study is therefore to evaluate robot-assisted hybrid esophagectomy in terms of perioperative course and complication rate and to compare the results with those of patients who underwent an open procedure. Hereby, we want to analyze whether the robotic hybrid approach is superior to the open procedure as already published for the full robotic approach. A second focus was to compare laparoscopic and robotic hybrid esophagectomy in order to answer the question whether the use of the robotic technique is justified in hybrid procedures and associated with further advantages for the affected patients.

## Material and methods

### Patient cohort

This is a retrospective analysis of a prospectively maintained database. For this study, all consecutive patients who had undergone en-bloc esophagectomy for a malignant tumor of the esophagus or the esophagogastric junction at the University Hospital Magdeburg, Germany, from 1 January 2010 to 1 March 2021 were considered for analysis. All patients had a resectable tumor without distant metastasis. All were thoroughly evaluated before their operation and were medically fit for the procedure. Patients with an adenocarcinoma or a squamous cell carcinoma who were diagnosed with a locally advanced tumor (uT3/4) were recommended to receive neoadjuvant therapy.

For the present study, patients were only considered for analysis when they had undergone esophagectomy during the study period using the open or the hybrid technique (abdominal part: laparoscopic or robotic; thoracic part: open). In addition, patients were included only when they had received a circular stapled intrathoracic anastomosis. Therefore, exclusion criteria were as follows: limited resection; transhiatally extended gastrectomy; en-bloc esophagectomy with cervical anastomosis and intrathoracic anastomosis: handsewn or with linear stapler.

### Parameters

The following demographic, clinical, surgery-, and tumor-related parameters were used in this study:

- Demographic and clinical parameters: age (years), gender, body mass index (BMI, kg/m^2^), pre-existing comorbidities, ASA classification, hospital length of stay (LOS) (days), postoperative ICU stay (days), total ICU stay (days).
- Surgery- and tumor-associated parameters: intraoperative complications, operation duration (minutes), type of malignancy (adenocarcinoma, AEG; squamous cell carcinoma, SCC; others), neoadjuvant therapy, number of lymph nodes retrieved, postoperative resection status (R-status).

Postoperative complications:

- *Procedure-related complications*: anastomotic leak, chylothorax, delayed gastric emptying (DGE), postoperative hiatal hernia, recurrent laryngeal nerve palsy, wound complications.
- General complications: pulmonary complications, cardiac complications, postoperative sepsis, postoperative delirium, 30-day mortality.

### Surgical technique

Conventional laparoscopic hybrid esophagectomy is performed using a five 12-mm trocar technique. The procedure starts with a complete gastrolysis and the formation of a 3-cm-wide gastric conduit. This is followed by abdominal lymphadenectomy and transhiatal dissection. During the thoracic part (open approach), the esophagus is completely mobilized. The lymphadenectomy routinely comprises the paraesophageal and the infracarinal lymph nodes. The paratracheal lymph nodes are removed in all patients with squamous cell carcinoma, in patients with adenocarcinoma only if they are enlarged or if tumorous infiltration is suspected (on CT scan or on intraoperative finding). For the intrathoracic end-to-side esophagogastrostomy, a circular stapler (25 or 28 French) is used. In November 2018, robot-assisted esophagectomy was implemented in our department using the Da Vinci Si platform. Since July 2019, procedures have been performed using the Xi system (Intuitive Surgical Inc., Sunnyvale, CA). Robot-assisted hybrid esophagectomy is performed based on the same standardized technique as in the conventional laparoscopic approach using three 8-mm, two 12-mm trocars, and a liver retractor.

### Statistical analysis

All analyses were conducted using SAS 9.4 (SAS Institute Inc., Cary, NC, USA) and deliberately reviewed to the full level of significance of alpha = 0.05. Thus, no adjustment for multiple testing was applied and each *p* value ≤ 0.05 was considered to correspond to a significant result in an explorative sense. *p* values < 0.1 were interpreted as a trend. In order to gain an initial overview, a univariate analysis of the sample with respect to the surgical technique was performed. The robust *t*-test (Satterthwaite) was used for continuous outcome parameters (log-transformed, if applicable); Fisher’s exact test was used for categorical outcome parameters. Analyses were conducted on all available data as observed, i.e., no imputation of missing values. Since the number of available patients was too low for adequate multivariable modeling, a propensity score matching was applied in order to compare balanced matched samples. The propensity scores were gained via logistic regression using the following matching variables to adjust the comparison of the robot-assisted hybrid and the open technique: age, BMI, gender, ASA class, and type of malignancy. Since the number of available cases for comparison of the laparoscopic and the robot-assisted hybrid technique was even lower, the matching variables were reduced to age, BMI, and gender. Both propensity score matchings were restricted to patients with SCC and AEG type I/II. The robust greedy algorithm was used for matching applying a caliper of 2 standard deviations. Matched pairs were analyzed for systematic differences using the McNemar test and the paired *t*-test for categorical and continuous outcome variables, respectively.

## Results

### Patient cohort

In total, 227 patients were included in this study. Thereof, 60.4% (*n* = 137) had undergone open esophagectomy. The laparoscopic hybrid technique was applied in 19.8% (*n* = 45), the robotic hybrid technique in 19.8% (*n* = 45). Eighty-one patients (37.5%) were diagnosed with SCC, 139 patients (61.2%) with AEG type I and II. The male gender predominated with 89.0%. One hundred forty patients (61.7%) were classified as ASA class 1 and 2. The majority of the patients (*n* = 117, 78%) had undergone neoadjuvant therapy. The mean operation time was 343 min. Intraoperative problems occurred in 29 cases (12.8%). R0 resection was achieved in 211 patients (93.0%). The mean number of harvested lymph nodes was 23.3. In total, 131 patients developed postoperative complications, most frequently pulmonary complications being: pneumothorax: *n* = 41, 18%; pneumonia: *n* = 40, 17.6%; pleural empyema: *n* = 19, 8.4%; DGE: *n* = 69, 30.4%; postoperative delirium: *n* = 36, 15.8%; chylothorax: *n* = 34, 15%; and sepsis: *n* = 27, 11.9%. Anastomotic leak occurred in 39 patients (17.2%). The overall morbidity rate was 57.7%; the 30-day mortality rate was 2.6%. The mean postoperative ICU stay lasted 3.6 days; the mean hospital LOS was 19.7 days.

### Univariate analysis comparing all surgical techniques

The univariate analysis of all surgical techniques (open esophagectomy, laparoscopic hybrid, robotic hybrid) revealed no significant difference regarding age, gender, preoperative BMI, frequency of neoadjuvant therapy, ASA classification, or pre-existing comorbidities (Table 1). In contrast, significant differences regarding the frequency of intraoperative and postoperative complications were found (*p* < 0.01), mostly for the following parameters: anastomotic leak (*p* = 0.02), postoperative pneumothorax (*p* = 0.04), acute respiratory distress syndrome (*p* = 0.01), and postoperative wound complications (*p* < 0.01) (Tables 2 and 3). All these complications were more frequently diagnosed in the open surgery group. Additionally, there was a significant difference in the number of lymph nodes harvested, with a higher number in patients who had undergone minimally invasive surgery (*p* < 0.01) (Table 2). Furthermore, there were significant differences between all techniques regarding the mean ICU and hospital length of stay (*p* < 0.01). Patients who had undergone minimally invasive hybrid esophagectomy had a shorter postoperative and total ICU stay as well as a shorter hospital length of stay (Table 1). By contrast, the mean operation time differed significantly between all techniques, with the longest duration in the robotic hybrid group (*p* < 0.01) (Table 2).

**Table 1:** Univariate analysis of demographic and clinical parameters: comparison of the open, the laparoscopic, and the robotic hybrid technique (the robust *t*-test (Satterthwaite) was used for continuous outcome parameters, Fisher’s exact test was used for categorical outcome parameters)

		Open		Laparoscopic hybrid		Robotic hybrid		*p*
		*n*	%	*n*	%	*n*	%	
**Gender**	Male	122	89.1	41	91.1	39	86.7	0.87
Age (years) *mean [*± *STD]*		63.7 *[*± *9.9]*		63.2 *[*± *11.6]*		61.8 *[*± *8.8]*		0.55
BMI (kg/m^2^) *mean [*± *STD]*		25.7 *[*± *4.6]*		25.9 *[*± *5.1]*		27.2 *[*± *6.6]*		0.23
ASA class	1–2	87	64.9	26	60.5	27	60.0	0.77
	3–4	47	35.1	17	39.5	18	40.0	
Type of malignancy	SCC	54	39.4	14	31.1	13	28.9	n.a
	AEG type I/II	81	58.4	28	62.2	31	68.9	
Pre-existing comorbidity	Cardiac	81	59.6	22	48.9	20	44.4	0.14
	Pulmonary	43	31.6	12	26.7	12	26.7	0.77
	Vascular	48	36.1	14	31.1	12	28.6	0.63
	Hepatic	21	15.4	3	6.7	8	17.8	0.28
	Renal	11	8.1	6	13.3	5	11.1	0.51
	GERD	48	35.6	11	25.0	14	33.3	0.45
	Barrett’s esophagus	47	35.1	14	31.8	18	41.9	0.59
	Smoking	79	59.0	29	64.4	20	44.4	0.13
Hospital stay (days) *mean [range]*		23.9 *[22.3; 25.6]*		19.7 *[17.8; 21.6]*		15.5 *[13.8; 17.1]*		< 0.01
Postoperative ICU stay (days) *mean [min; max]*		4.6 *[2.2; 7.1]*		4.1 *[2.1; 6.2]*		2.3 *[0.2; 4.3]*		< 0.01
Total ICU stay (days) *mean [min; max]*		6 *[3.3; 8.8]*		4.7 *[2.5; 6.8]*		2.6 *[0.3; 5.0]*		< 0.01

*STD* standard deviation, *BMI* body mass index, *ASA* American Society of Anesthesiologists, *GERD* gastroesophageal reflux disease, *SCC* squamous cell carcinoma, *AEG* adenocarcinoma of the esophagogastric junction, *ICU* intensive care unit

**Table 2:** Univariate analysis of surgery- and tumor-associated parameters: comparison of the open, the laparoscopic, and the robotic hybrid technique (the robust *t*-test (Satterthwaite) was used for continuous outcome parameters, Fisher’s exact test was used for categorical outcome parameters)

		Open		Laparoscopic hybrid		Robotic hybrid		*p*
		*n*	%	*n*	%	*n*	%	
Operation duration (minutes) *mean [± STD]*		314* [± 69.6]*		320 *[± 59.7]*		395 *[± 57.0]*		< 0.01
Intraoperative complications		22	24.4	6	14.3	1	2.3	< 0.01
Number of LN retrieved *mean [min; max]*		20.4* [18.9; 22.0]*		23.1* [21.5; 24.7]*		26.3 *[24.8; 27.7]*		< 0.01
Nodal status	N0	72	53.3	24	54.5	26	59.1	0.88
	N1	32	23.7	13	29.5	8	18.2	
	N2	19	14.1	4	9.1	8	18.2	
	N3	11	8.1	3	6.8	2	4.6	
L1 status		45	34.1	16	35.6	17	40.5	0.72
V1 status		17	13.0	9	20.5	9	21.4	0.26
Pn1 status		28	21.4	14	32.6	15	35.7	0.10
R0 status		126	93.3	43	97.7	42	95.5	0.71

*STD* standard deviation, *LN* lymph nodes

**Table 3:** Univariate analysis of postoperative complications: comparison of the open, the laparoscopic, and the robotic hybrid technique (Fisher’s exact test was used)

	Open		Laparoscopic hybrid		Robotic hybrid		*p*
	*n*	%	*n*	%	*n*	%	
Pneumothorax	31	22.6	7	15.6	3	6.7	0.04
Pneumonia	30	22.1	5	11.1	5	11.1	0.12
ARDS	17	12.5	2	4.4	0	0.0	0.01
Postoperative sepsis	20	14.6	4	8.9	3	6.7	0.33
Postoperative delirium	24	17.5	3	6.7	9	20.0	0.14
Wound complication	22	16.1	2	4.4	0	0.0	< 0.01
Cardiac complication	17	12.4	4	8.9	3	6.7	0.57
Anastomotic leak	31	22.6	5	11.4	3	6.7	0.03
Chylothorax	21	15.4	6	13.3	7	15.6	0.97
DGE	55	40.4	12	28.6	2	4.4	< 0.01
RLNP	2	1.5	1	2.2	0	0.0	1.00
30-day mortality	4	2.9	2	4.4	0	0.0	0.44

*ARDS* acute respiratory distress syndrome, *DGE* delayed gastric emptying, *RLNP* recurrent laryngeal nerve palsy

### Propensity score matching analysis: comparison of the open and the robotic hybrid technique

In order to compare the open and the robotic hybrid technique, a 1:1 propensity score matching (PSM) was performed. The following matching variables were chosen for the PSM analysis: gender, age, BMI, ASA class, and type of malignancy. PSM of the initial group resulted in two equal groups of 42 patients. The robotic hybrid technique was associated with a significantly lower rate of both, intraoperative complications (*p* = 0.03) and postoperative morbidity (*p* < 0.01). In particular, patients who had undergone open esophagectomy more frequently developed a postoperative pneumothorax (*p* = 0.01) and DGE (*p* < 0.01) (Table 4). The lymph node yield was significantly higher in the robotic hybrid group (*p* = 0.048). In addition, the mean hospital LOS (*p* < 0.01) and the postoperative ICU stay (*p* < 0.01) were significantly shorter in the robotic hybrid group (Table 5). The use of the robotic hybrid approach was also associated with a lower anastomotic leak rate (*p* = 0.09), a lower occurrence of postoperative sepsis (*p* = 0.07), and less pleural effusions (*p* = 0.09). However, these differences were not statistically significant. By contrast, the mean operation time was significantly longer in the robotic group (*p* < 0.01) (Table 5).

**Table 4:** Results of analysis after propensity score matching for categorical parameters; non-diagonal elements (disadvantages) reflect the discordant cases within pair (open vs. robotic hybrid technique), matched pairs were analyzed for systematic differences using McNemar test; only parameters with *p* < 0.3 are shown

	Disadvantages		*p*
	Open (*n* = 42)	Robotic (*n* = 42)	
Intraoperative complications (+ 3.7% concordant cases)	6/27 (22.2%)	0/27 (0%)	0.03
Postoperative complications (+ 23.8% concordant cases)	20/42 (47.6%)	4/42 (9.5%)	< 0.01
Anastomotic leak (0% concordant cases)	10/42 (23.8%)	3/42 (7.1%)	0.09
Delayed gastric emptying (0% concordant cases)	17/42 (40.5%)	2/42 (4.8%)	< 0.01
Pleural effusion (+ 2.4% concordant cases)	10/42 (23.8%)	3/42 (7.1%)	0.09
Pneumothorax (0% concordant cases)	14/42 (33.3%)	3/42 (7.1%)	0.01
Postoperative sepsis (+ 2.4% concordant cases)	7/42 (16.7%)	1/42 (2.4%)	0.07

**Table 5:** Results of analysis after propensity score matching for continuous parameters (*n* = 42 pairs, open vs. robotic hybrid technique, matched pairs were analyzed for systematic differences using the paired *t*-test); only parameters with *p* < 0.3 are shown

*n* = 42	Mean (open – robotic)	Lower limit of mean	Upper limit of mean	*p*
Hospital length of stay (days)	12.1	4.6	19.6	< 0.01
Postoperative ICU stay (days)	4.7	2.0	7.4	< 0.01
Total ICU stay (days)	9.1	3.7	14.6	< 0.01
Operation duration (minutes)	− 85.8	− 117.0	− 54.5	< 0.01
Number of LN retrieved	− 5.1	− 10.2	− 0.04	0.048

*ICU* intensive care unit, *LN* lymph nodes

### Propensity score matching analysis: comparison of the laparoscopic and the robotic hybrid technique

The following matching variables were chosen for the PSM analysis comparing the laparoscopic and the robotic hybrid technique: age, gender, and ASA class. In total, two equal groups of 35 patients were analyzed. The number of patients who experienced DGE during the postoperative course was significantly lower in the robotic hybrid group (*p* = 0.02) (Table 6). Additionally, the hospital LOS was shorter in the robotic group. However, this difference was not statistically significant (*p* = 0.08). By contrast, the operation time was significantly longer in the robotic hybrid group (*p* < 0.01) (Table 7).

**Table 6:** Results of analysis after propensity score matching for categorical parameters; non-diagonal elements (disadvantages) reflect the discordant cases within pair (laparoscopic vs. robotic hybrid), matched pairs were analyzed for systematic differences using McNemar test; only parameters with *p* < 0.3 are shown

	Disadvantages		*p*
	Laparoscopic (*n* = 35)	Robotic (*n* = 35)	
Postoperative complications (+ 22.9% concordant cases)	12/35 (34.3%)	6/35 (17.1%)	0.24
Delayed gastric emptying (0% concordant cases)	9/33 (27.3%)	1/33 (3.0%)	0.02
Pleural effusion (0% concordant cases)	9/35 (25.7%)	4/35 (11.4%)	0.27
Postoperative delirium (+ 2.9% concordant cases)	2/35 (5.7%)	7/36 (20.0%)	0.18

**Table 7:** Results of analysis after propensity score matching for continuous parameters (*n* = 35 pairs; laparoscopic vs. robotic hybrid, matched pairs were analyzed for systematic differences using the paired *t*-test); only parameters with *p* < 0.3 are shown

*n* = 35	Mean (laparoscopic – robotic)	Lower limit of mean	Upper limit of mean	*p*
Hospital length of stay (days)	6.1	− 0.9	13.2	0.08
Postoperative ICU stay (days)	2.7	− 0.9	6.3	0.13
Total ICU stay (days)	2.5	− 1.2	6.1	0.18
Operation duration (minutes)	− 68.7	− 99.2	− 38.2	< 0.01

## Discussion

En-bloc esophagectomy represents one of the most complex surgical procedures. Such operations remain associated with a high postoperative complication rate even today. The introduction of minimally invasive surgery has resulted in a significant reduction of postoperative morbidity and mortality compared to open surgery and has also significantly improved the outcome for the affected patients. Initial randomized controlled trials demonstrated a reduction of postoperative complications following minimally invasive esophagectomy [8, 9]. This finding was confirmed in a meta-analysis of randomized controlled trials published in 2021 comparing minimally invasive to open esophagectomy in patients with esophageal cancer [14]. The authors showed that the minimally invasive approach is associated with fewer postoperative complications, in particular, fewer pulmonary complications. However, survival was comparable for both techniques.

During the last decade, robot-assisted techniques have gained tremendous popularity in the field of surgery. For en-bloc esophagectomy, multiple advantages of the full robot-assisted approach compared to open esophagectomy were demonstrated in a randomized controlled study (ROBOT trial) [10, 11]. Interestingly, there are nearly no data available as to whether the use of robotic assistance in hybrid esophagectomy (robotic abdominal part) is efficient and beneficial and will thus provide advantages despite the higher procedural costs. Therefore, the first aim of the present study was to evaluate robot-assisted hybrid esophagectomy and to compare this approach to open esophagectomy. Our aim here was to analyze whether the findings of previous studies—demonstrating advantages of robotic assistance—can be confirmed also for the robotic hybrid approach. For the present analysis, we included all consecutive patients who had undergone transthoracic en-bloc esophagectomy with intrathoracic circular stapled anastomosis for a malignant tumor of the esophagus or the esophagogastric junction.

The results of the analysis after propensity score matching comparing the robotic hybrid with the open approach showed a significant reduction of the intraoperative complication rate and of postoperative morbidity in favor of the robotic technique. In addition, the use of robotic assistance was associated with a significantly shorter ICU and hospital LOS and with a higher median number of lymph nodes retrieved. By contrast, the robotic procedure was associated with a significantly longer operation time. In conclusion, the findings of the present analysis confirm the results of previous studies. A multi-center randomized controlled trial (MIRO trial) compared minimally invasive hybrid esophagectomy (laparoscopy/thoracotomy) with the open procedure [9]. The 3-year follow-up of a total of 207 patients showed a significantly lower postoperative morbidity in the hybrid group (35.9% vs. 64.4%), especially less pulmonary complications [15]. A further analysis compared the 5-year survival but failed to show any significant difference between the groups. Nevertheless, the occurrence of postoperative complications, especially pulmonary complications, was associated with a lower survival rate [16]. The ROBOT trial compared robotic and open en-bloc esophagectomy. One of the primary endpoints was the postoperative complication rate (Clavien-Dindo ≥ 2) [10]. The study confirmed significant advantages of the robot-assisted technique. The robotic group showed a lower percentage of pulmonary and cardiac complications. Furthermore, the intraoperative blood loss and the postoperative pain were significantly reduced. The oncologic outcome was equivalent. In contrast to our results, the ICU and hospital LOS did not differ significantly between the groups [17]. In contrast to our study, all patients in the ROBOT trial received a cervical anastomosis and the thoracic part was also performed with robotic assistance. To the best of our knowledge, there is only one retrospective study comparing robot-assisted hybrid (robotic abdominal part) and open esophagectomy [18]. The authors compared 160 open with 56 robot-assisted hybrid procedures. It was demonstrated that the hybrid approach was associated with a significantly higher lymph node harvest and a shorter hospital stay which is in line with the results of our study. Furthermore, it was shown that patients that had undergone a hybrid procedure experienced a significantly lower intraoperative blood loss and fewer grade 2 or higher complications during the postoperative course than patients in the open group. In contrast to our study, no propensity score matching analysis was performed.

Several recent studies have demonstrated that robotic assistance may further improve patients’ outcomes when compared to the conventional minimally invasive approach [12, 13]. However, most of these studies compared the full robotic esophagectomy with the total minimally invasive approach using the conventional laparoscopic/thoracoscopic technique. There is nearly no evidence of whether the use of robotic assistance in hybrid esophagectomy (minimally invasive abdominal part) is beneficial for the affected patients. In the second part of the present study, laparoscopic hybrid and robotic hybrid en-bloc esophagectomy were compared using a propensity score matching analysis. We were able to show that the robotic hybrid technique was associated with a significantly lower rate of postoperative DGE but also with a significantly longer operation time.

A Chinese prospective multi-center study (RAMIE trial) compared the effectiveness and safety of robot-assisted versus conventional minimally invasive esophagectomy [12]. In contrast to our study, the operation time was significantly shorter and the thoracic lymph node harvest in patients after neoadjuvant therapy was higher in the robotic group. The overall postoperative complication rate, postoperative pulmonary complications, the rate of anastomotic leaks and of recurrent laryngeal nerve palsies were similar in both groups. The occurrence of postoperative DGE was not analyzed in this study. A German single-center study compared 50 patients who had undergone total minimally invasive and 50 patients who had undergone full robotic esophagectomy. The use of the robotic technique resulted in a significant reduction of the ICU stay as well as a higher lymph node harvest [13]. These results were not confirmed by our study. However, it should be noted that both studies compared the full robotic with the conventional total minimally invasive approach.

There is currently only one published study comparing robotic and conventional minimally invasive hybrid esophagectomy (laparoscopy/thoracotomy). In this study, 44 patients who had undergone laparoscopic hybrid esophagectomy were compared to 44 patients who had undergone robotic hybrid surgery. As in our study, all patients received an intrathoracic circular stapled anastomosis. No significant differences regarding general or surgical complications (i.e., anastomotic leak, pneumonia, chylothorax) were found between the groups. Likewise, both groups had a similar oncological outcome (lymph node yield, R0 resection rate) and a comparable ICU and hospital LOS. The median operation time was longer in the robotic hybrid group. In contrast to our study, however, this result was not statistically significant [19]. Interestingly, our study identified a significantly lower DGE rate in the robotic hybrid group. This complication was not analyzed in the study by Giulini et al. A potential explanation for the lower DGE rate in the robotic group might be the lesser gastric trauma experienced during the abdominal part, both during gastric mobilization and during creation of the gastric conduit. What contradicts this theory is that the same open approach was used during the thoracic part, especially the same anastomotic technique was used in both groups. For DGE following transthoracic en-bloc esophagectomy, several risk factors have been described previously. One study identified female gender, pre-existing pulmonary comorbidity, anastomotic leaks, and postoperative pulmonary complications as risk factors for the occurrence of DGE following esophagectomy [20]. Another study by Babic et al. also demonstrated female gender as a risk factor for DGE. However, a significant association between anastomotic leaks and the occurrence of DGE was not confirmed. None of the studies was able to show any influence of the surgical technique on the development of DGE following esophagectomy [21]. Thus, we are not able to provide any final sufficient explanation for this finding of our study.

There are a few limitations of the present study that have to be acknowledged. Firstly, the retrospective and single-center design. Secondly, the relatively low number of patients, particularly after propensity score matching. The results of the study may also have been influenced by the long study period including different surgeons. However, all surgeons have extensive experience in upper GI and esophageal surgery. Furthermore, the effect of the learning curve after implementation of the robotic program may also have influenced our results.

## Conclusion

The use of minimally invasive techniques significantly reduces the postoperative complication rate in patients undergoing transthoracic en-bloc esophagectomy. This was demonstrated for both total minimally invasive and hybrid procedures. The use of robotic assistance was also shown to be superior when compared to open esophagectomy. In the present study, this was confirmed also for the robotic hybrid technique. By contrast, the comparison of the laparoscopic and the robotic hybrid approach revealed only few advantages for the robotic technique. Further studies are now needed in order to evaluate which surgical technique—currently available for patients undergoing en-bloc esophagectomy—is the most beneficial for the affected patients. Here, the future role of hybrid techniques compared to total minimally invasive techniques needs to be determined. Furthermore, the question whether the use of cost-intensive robotic techniques further improves postoperative morbidity and mortality as well as the oncological outcome has to be answered.

## Data Availability

Data and material are available at frank.benedix@med.ovgu.de.

## References

[1] Sung H, Ferlay J, Siegel RL, Laversanne M, Soerjomataram I, Jemal A, Bray F (2021). Global cancer statistics 2020: GLOBOCAN estimates of incidence and mortality worldwide for 36 cancers in 185 countries. CA Cancer J Clin.

[2] Uhlenhopp DJ, Then EO, Sunkara T (2020). Epidemiology of esophageal cancer: update in global trends, etiology and risk factors. Clin J Gastroenterol.

[3] Turati F, Tramacere I, La Vecchia C, Negri E (2013). A meta-analysis of body mass index and esophageal and gastric cardia adenocarcinoma. Ann Oncol.

[4] Hazelton WD, Curtius K, Inadomi JM, Vaughan TL, Meza R, Rubenstein JH, Hur C, Luebeck EG (2015). The role of gastroesophageal reflux and other factors during progression to esophageal adenocarcinoma. Cancer Epidemiol Biomarkers Prev.

[5] Leitlinienprogramm Onkologie (Deutsche Krebsgesellschaft, Deutsche Krebshilfe, AWMF): S3-Leitlinie Diagnostik und Therapie der Plattenepithelkarzinome und Adenokarzinome des Ösophagus, Langversion 3.0, Oktober 2021, AWMF Registernummer: 021/023OL https://www.leitlinienprogramm-onkologie.de/leitlinien/oesophaguskarzinom/ (abgerufen am: 22.03.2022)

[6] Kuppusamy MK (2022). Low DE; International Esodata Study Group (IESG) Evaluation of international contemporary operative outcomes and management trends associated with esophagectomy: a 4-year study of >6000 patients using ECCG definitions and the online Esodata database. Ann Surg.

[7] Hildebrand P, Roblick UJ, Keller R, Kleemann M, Mirow L, Bruch HP (2007). Was bringt die Minimalisierung des Zugangstraumas für den Patienten?. Chirurg.

[8] Biere SS, Maas KW, Bonavina L, Garcia JR, van Berge Henegouwen MI, Rosman C, Sosef MN, de Lange ES, Bonjer HJ, Cuesta MA, van der Peet DL. Traditional invasive vs. minimally invasive esophagectomy: a multi-center, randomized trial (TIME-trial). BMC Surg. 2011;11:2. 10.1186/1471-2482-11-2 10.1186/1471-2482-11-2 PMC3031195 21226918

[9] Briez N, Piessen G, Bonnetain F (2011). Open versus laparoscopically-assisted oesophagectomy for cancer: a multicentre randomised controlled phase III trial - the MIRO trial. BMC Cancer.

[10] van der Sluis PC, Ruurda JP, van der Horst S, Verhage RJ, Besselink MG, Prins MJ, Haverkamp L, Schippers C, Rinkes IH, Joore HC, Ten Kate FJ, Koffijberg H, Kroese CC, van Leeuwen MS, Lolkema MP, Reerink O, Schipper ME, Steenhagen E, Vleggaar FP, Voest EE, Siersema PD, van Hillegersberg R (2012). Robot-assisted minimally invasive thoraco-laparoscopic esophagectomy versus open transthoracic esophagectomy for resectable esophageal cancer, a randomized controlled trial (ROBOT trial). Trials.

[11] de Groot EM, van der Horst S, Kingma BF, Goense L, van der Sluis PC, Ruurda JP, van Hillegersberg R (2020). Robot-assisted minimally invasive thoracolaparoscopic esophagectomy versus open esophagectomy: long-term follow-up of a randomized clinical trial. Dis Esophagus.

[12] Yang Y, Li B, Yi J, Hua R, Chen H, Tan L, Li H, He Y, Guo X, Sun Y, Yu B, Li Z. Robot-assisted Versus Conventional Minimally Invasive Esophagectomy for Resectable Esophageal Squamous Cell Carcinoma: Early Results of a Multicenter Randomized Controlled Trial: the RAMIE Trial. Ann Surg. 2022;275(4):646–653. 10.1097/SLA.0000000000005023 10.1097/SLA.0000000000005023 34171870

[13] Tagkalos E, Goense L, Hoppe-Lotichius M, Ruurda JP, Babic B, Hadzijusufovic E, Kneist W, van der Sluis PC, Lang H, van Hillegersberg R, Grimminger PP (2020). Robot-assisted minimally invasive esophagectomy (RAMIE) compared to conventional minimally invasive esophagectomy (MIE) for esophageal cancer: a propensity-matched analysis. Dis Esophagus.

[14] Müller-Stich BP, Probst P, Nienhüser H, Fazeli S, Senft J, Kalkum E, Heger P, Warschkow R, Nickel F, Billeter AT, Grimminger PP, Gutschow C, Dabakuyo-Yonli TS, Piessen G, Paireder M, Schoppmann SF, van der Peet DL, Cuesta MA, van der Sluis P, van Hillegersberg R, Hölscher AH, Diener MK, Schmidt T (2021). Meta-analysis of randomized controlled trials and individual patient data comparing minimally invasive with open oesophagectomy for cancer. Br J Surg.

[15] Mariette C, Markar SR, Dabakuyo-Yonli TS, Meunier B, Pezet D, Collet D, D'Journo XB, Brigand C, Perniceni T, Carrère N, Mabrut JY, Msika S, Peschaud F, Prudhomme M, Bonnetain F, Piessen G; Fédération de Recherche en Chirurgie (FRENCH) and French Eso-Gastric Tumors (FREGAT) Working Group. Hybrid Minimally Invasive Esophagectomy for Esophageal Cancer. N Engl J Med. 2019;380(2):152–162. 10.1056/NEJMoa1805101 10.1056/NEJMoa1805101 30625052

[16] Nuytens F, Dabakuyo-Yonli TS, Meunier B, Gagnière J, Collet D, D'Journo XB, Brigand C, Perniceni T, Carrère N, Mabrut JY, Msika S, Peschaud F, Prudhomme M, Markar SR, Piessen G; Fédération de Recherche en Chirurgie (FRENCH) and French Eso-Gastric Tumors (FREGAT) Working Groups (2021). Five-year survival outcomes of hybrid minimally invasive esophagectomy in esophageal cancer: results of the MIRO randomized clinical trial. JAMA Surg.

[17] van der Sluis PC, van der Horst S, May AM, Schippers C, Brosens LAA, Joore HCA, Kroese CC, Haj Mohammad N, Mook S, Vleggaar FP, Borel Rinkes IHM, Ruurda JP, van Hillegersberg R (2019). Robot-assisted minimally invasive thoracolaparoscopic esophagectomy versus open transthoracic esophagectomy for resectable esophageal cancer: a randomized controlled trial. Ann Surg.

[18] Rolff HC (2017). Robot-assisted hybrid esophagectomy is associated with a shorter length of stay compared to conventional transthoracic esophagectomy: a retrospective study. Minim Invasive Surg.

[19] Giulini L, Nasser CA, Tank J, Papp M, Stein HJ, Dubecz A (2021). Hybrid robotic versus hybrid laparoscopic Ivor Lewis oesophagectomy: a case-matched analysis. Eur J Cardiothorac Surg.

[20] Benedix F, Willems T, Kropf S, Schubert D, Stübs P, Wolff S (2017). Risk factors for delayed gastric emptying after esophagectomy. Langenbecks Arch Surg.

[21] Babic B, Schiffmann LM, Fuchs HF, Mueller DT, Schmidt T, Mallmann C, Mielke L, Frebel A, Schiller P, Bludau M, Chon SH, Schroeder W, Bruns CJ. There is no correlation between a delayed gastric conduit emptying and the occurrence of an anastomotic leakage after Ivor-Lewis esophagectomy. Surg Endosc. 2022;36(9):6777–6783. 10.1007/s00464-021-08962-5 10.1007/s00464-021-08962-5 PMC9402722 34981236

